# High Tolerance of *Hydrogenothermus marinus* to Sodium Perchlorate

**DOI:** 10.3389/fmicb.2017.01369

**Published:** 2017-07-18

**Authors:** Kristina Beblo-Vranesevic, Harald Huber, Petra Rettberg

**Affiliations:** ^1^Radiation Biology Division, Institute of Aerospace Medicine, German Aerospace Center (DLR e.V.) Cologne, Germany; ^2^Institute for Microbiology and Archaea Center, Faculty of Biology and Preclinical Medicine, University of Regensburg Regensburg, Germany

**Keywords:** perchlorates, morphology, thermophiles, desiccation, survival

## Abstract

On Mars, significant amounts (0.4–0.6%) of perchlorate ions were detected in dry soil by the Phoenix Wet Chemistry Laboratory and later confirmed with the Mars Science Laboratory. Therefore, the ability of *Hydrogenothermus marinus*, a desiccation tolerant bacterium, to survive and grow in the presence of perchlorates was determined. Results indicated that *H. marinus* was able to tolerate concentrations of sodium perchlorate up to 200 mM (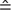 1.6%) during cultivation without any changes in its growth pattern. After the addition of up to 440 mM (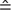 3.7%) sodium perchlorate, *H. marinus* showed significant changes in cell morphology; from single motile short rods to long cell chains up to 80 cells. Furthermore, it was shown that the known desiccation tolerance of *H. marinus* is highly influenced by a pre-treatment with different perchlorates; additive effects of desiccation and perchlorate treatments are visible in a reduced survival rate. These data demonstrate that thermophiles, especially *H. marinus*, have so far, unknown high tolerances against cell damaging treatments and may serve as model organisms for future space experiments.

## Introduction

Research on perchlorates has increased over the course of the last 30 years. The natural occurrence of perchlorates is in geographically limited very arid environments such as the Atacama Desert in Chile (Trumpolt et al., 2005; Catling et al., 2010). For example, perchlorate concentrations in nitrate mineral deposits in the Atacama Desert are known to be 0.028 wt % (Michalski et al., 2004).

Not only on Earth, but also on our neighboring planet Mars, perchlorates have been detected, however, in much higher concentrations than on Earth. Significant amounts of perchlorate ions (0.4–0.6%) were first detected by the Phoenix lander in regolith in the northern polar region (Vastitas Borealis) (Hecht et al., 2009). Data from additional studies provide strong evidence that the soil samples analyzed by the Wet Chemical Laboratory on board the Phoenix lander contain calcium perchlorate (Ca-perchlorate) and magnesium perchlorate (Mg-perchlorate) (Kounaves et al., 2014). In general, the data obtained from the Mars Science Laboratory suggests that perchlorates occur throughout the entire surface of Mars (Archer et al., 2013). At distinct craters, different perchlorates were identified. Data from the Compact Reconnaissance Imaging Spectrometer on board the Mars Reconnaissance Orbiter detected a mixture of sodium perchlorate (Na-perchlorate) and Mg-perchlorate in the Palikir and Hale crater (Ojha et al., 2015), at Horowitz crater, Na-perchlorate can be found (Ojha et al., 2015) and Ca-perchlorate at the Gale Crater (Glavin et al., 2013).

Although the detailed formation mechanism is still unknown, two different formation mechanisms of Martian perchlorates have been proposed to explain their occurrence on the surface of Mars. One explanation suggests that the perchlorates were produced on the surface where Martian surface minerals catalyze the photochemical oxidation of chlorides to perchlorates (Schuttlefield et al., 2011; Kim et al., 2013). It was shown that in chloride containing Martian soil simulants, perchlorates are produced in the presence of ultraviolet light (Carrier and Kounaves, 2015). Another formation mechanism proposes atmospheric formations where Martian perchlorates may have originated are the result of reactivity of atmospheric oxidants in the arid environment on Mars (Catling et al., 2010).

The assessment of the potential habitability of Mars is based on one hand on the state of knowledge of the prevailing environmental conditions on the surface and in the subsurface of Mars and on the other hand on the state of knowledge of the survivability of Earth organisms under extreme Mars-like conditions (Cockell et al., 2016). Potential organisms should survive an exposure to perchlorates or should even able to live in the presence of perchlorates. Since the detection of perchlorates on Mars some years ago, the tolerance of non-perchlorate metabolizing microorganisms against perchlorates has been tested (e.g., Oren et al., 2014; Shcherbakova et al., 2015; Kral et al., 2016; Al Soudi et al., 2017). In general, perchlorate tolerant microorganisms can be affiliated in two groups: organisms that are tolerant to perchlorates without metabolizing them and perchlorate tolerant microorganisms that consume/metabolize perchlorates. Thereby, the perchlorate (ClO_4_^-^) is reduced in three steps to chloride (Cl^-^); the reduction potential is *E*^0^ = 1.29 V for the couple ClO_4_^-^/Cl^-^ (Logan, 1998; Coates and Achenbach, 2004). More than 40 different strains, mostly belonging to the proteobacterial phylum are known to have the ability to metabolize perchlorates (Coates and Achenbach, 2004; Nerenberg, 2013). Up to now, only a few archaeal perchlorate metabolizing representatives have been identified, namely *Archaeoglobus fulgidus* and several species from the family *Halobacteriaceae* (Liebensteiner et al., 2013; Oren, 2014). Since the metabolizing rates were the focus of these studies, the microorganisms were tested under low metabolic meaningful perchlorate concentrations of up to 0.1 M (Attaway and Smith, 1993). Thereby, the highest metabolic activity is measured at concentrations between 0.02 and 0.04 M. In general, data for the absolute resistance of the strains against high perchlorate concentrations are often missing. In recent times, non-perchlorate metabolizing microorganisms were also tested in their tolerance against perchlorates. Amongst these Archaea and Bacteria, different methanogenic, halophilic, and acidophilic representatives with different tolerance levels could be found: one example of a sensitive organism is the acidophilic iron sulfur bacterium *Acidithiobacillus ferrooxidans*, known to be possibly able to grow under Mars-like geochemical conditions (Bauermeister et al., 2014). *A. ferrooxidans* cannot multiply in the presence of 0.022 M (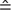 0.5%) and 0.044 M (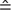 1%) Mg-perchlorate (Bauermeister, 2012). Different methanogenic Archaea (three *Methanobacterium* strains and two *Methanosarcina* strains) are also influenced in their growth behavior at low concentrations (up to 0.01 M) of Na-perchlorates (Shcherbakova et al., 2015). Spores of *Bacillus subtilis* germinate at a Na-perchlorate concentration of up to 0.1 M (Nagler and Moeller, 2015), a higher concentration (≥0.6 M) of Na-perchlorate and Mg-perchlorate lead to complete inactivation of the germination process (Nagler and Moeller, 2015). It has been reported that several halotolerant strains show only slight alterations in their growth pattern in the presence of perchlorates: nearly all of the halotolerant isolates grew in the presence of 0.05 M Mg-perchlorate. Some exhibited positive growth even in the presence 0.25 M Mg-perchlorate. While some growth may have occurred at levels as high as 0.5 M Mg-perchlorate for certain isolates, the data are limited and inconsistent (Al Soudi et al., 2017).

The identification of perchlorates at different locations on Mars directs the research interest to the investigation of perchlorate tolerance or even perchlorate metabolism in Earth organisms. Since on Earth, natural perchlorates occur mainly in arid areas like desserts, a first thought is that desiccation tolerant microorganisms might be capable to surviving exposures to perchlorates. In previous studies, a variety of Bacteria and Archaea have been screened for their tolerance to desiccation. Because of its desiccation tolerance, the deep-branching microaerophilic bacterium *Hydrogenothermus marinus* proved to be a promising model organism in many respects. *H. marinus* exhibits a unique tolerance to desiccation and to exposure to elevated salt concentrations (Beblo et al., 2009; Beblo-Vranesevic et al., 2017). Therefore, it was hypothesized that this organism would survive and grow in the presence of perchlorates. To test this hypothesis we determined the ability of this organism to replicate after exposure to different perchlorates, even in combination with desiccation. Additionally, the results from *H. marinus* were compared to other well known (model) microorganisms. These well studied organisms, namely *Escherichia coli*, *B. subtilis*, and *Deinococcus radiodurans*, originate from different habitats on Earth and are known to react in different ways to an exposure to cell damaging treatments.

## Materials and Methods

### Strain and Culture Conditions

*Hydrogenothermus marinus* DSM 12046^T^ was cultured in microoxic (2% O_2_ in 1 bar H_2_/CO_2_) modified VM1 medium (ZoBell, 1941; Stöhr et al., 2001). Incubated at optimal growth temperatures (*T*_opt._: 65°C), a cell density of approximately 10^8^ cells per ml was reached after 24 h of incubation. The reference strain, *E. coli* K12 wild-type (DSM No. 498), was cultivated in liquid NB medium (0.5% peptone, 0.3% meat extract) or plated on solidified NB medium (NB medium with 1.5% agar). Incubation temperature was at optimal growth temperature (*T*_opt._: 37°C). *B. subtilis* strain NCIB 3610^T^ (DSM No. 10) and *D. radiodurans* type strain R1^T^ (DSM No. 20539) were grown in TGY medium in liquid (0.5% tryptone, 0.3% yeast extract, 0.1% glucose) or were plated on solidified TGY medium (TGY medium with 1.5% agar). Incubation temperature was at organisms’ optimal growth temperature (*B. subtilis*: *T*_opt._: 37°C; *D. radiodurans*: *T*_opt._: 30°C). Additionally, purified *B. subtilis* spores were used for exposure experiments. Isolation of the spores was conducted according to Nagler et al. (2014).

### Exposure to Perchlorates

Different concentrations of three perchlorates [Na-perchlorate NaClO_4_; Mg-perchlorate Mg(ClO_4_)_2_; Ca-perchlorate Ca(ClO_4_)_2_] were added to cells grown at optimal standard conditions. Due to the limited solubility of the perchlorates, concentrations higher than 5 M could not be tested. The solubility of Na-perchlorate hydrate in water is 2090 g per liter at 15°C. But the solubility is noticeably reduced when the perchlorate is dissolved in the modified salty VM-1 medium.

During the shock experiments, the cells were exposed for a specified time (15 min or 96 h) to ascending concentrations of Na-perchlorate at ambient room temperature or at 65°C (only for *H. marinus*). Exposure was disrupted by a dilution step (1:100 and 1:400, respectively) and for analysis of the survivors; either the most probable number assay or standard spread-plate assay was used.

Additionally, *H. marinus* cells were cultivated in the presence of different concentrations of Na-perchlorate.

### Desiccation Experiments

Desiccation experiments were performed as described earlier (Beblo et al., 2009). Briefly, cell concentrations were determined by counting in a Thoma chamber (depth: 0.02 mm). The cell suspensions (2 ml) were spread evenly on four glass slides and dried for 24 h under oxic laboratory conditions (room temperature, average relative humidity 33 ± 3.5%).

### Determination of the Survival Rate

In general, growth and morphology of the cells was observed by phase-contrast light microscopy (Zeiss^®^ Axiolmager^TM^ M2) with 400× or 1000× magnification.

Desiccated cells on glass slides or non-desiccated control cells were transferred into a culture bottle with culture media. Perchlorate exposed *H. marinus* cells in liquid suspension were subjected to a dilution step (1:100). Since *H. marinus* do not form colonies on solid surfaces with sufficiently high efficiency (see strain references) plating assays on solid media could not be applied. Detection of viable cells was achieved by the most probable number technique via dilution series with 10-fold dilution steps (Franson, 1985).

For *E. coli*, *D. radiodurans*, and *B. subtilis* the stop reaction (1:400) dilution step was performed in sterile PBS buffer solution. Afterward further dilution series were prepared and an aliquot of each dilution was plated.

All experiments were performed at least three times independently, representing biological replicas. The data shown within the graphs represent mean values with standard deviations. The survival rate (S) was calculated as relative survival after treatment (*N*) compared to the non-treated control (*N*_0_) (*S* = *N*/*N*_0_). *D*_10_-values, giving the dose of perchlorates in Molar (M) which reduces the survival rate by one order of magnitude were calculated from the regression lines of the exponential slopes of the mean survival curves as described by Harm (1980).

## Results

**FIGURE 1:**
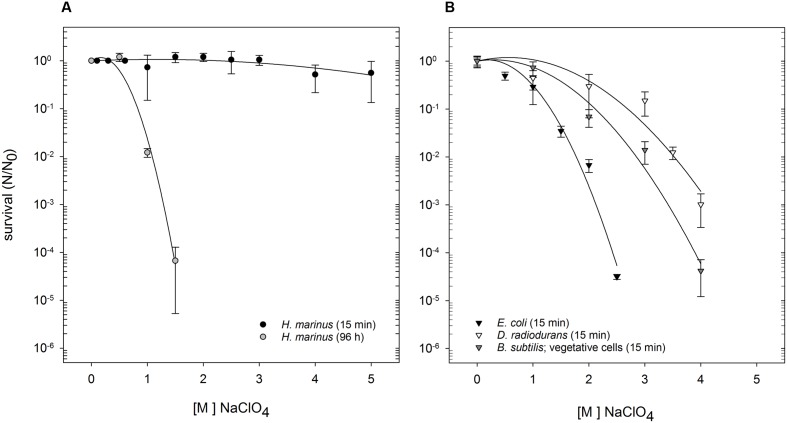
Survival of *H. marinus*, *E. coli*, *B. subtilis* (vegetative cells), and *D. radiodurans* after exposure to Na-perchlorate at room temperature. **(A)** *H. marinus* was either exposed to Na-perchlorate for 15 min (black circles) or for 96 h (gray circles). **(B)** *E. coli* (black triangle), *B. subtilis* (vegetative cells), and *D. radiodurans* were exposed for 15 min to Na-perchlorate.

Shock exposure (15 min) to Na-perchlorate did not change *H. marinus’* survivability, and can be deduced from the nearly straight line within the dose-survival curve (**Figure 1A**). This is true for concentrations up to 5 M, that corresponds to the maximal solubility limits of perchlorates in salty medium. After long term exposure (96 h), a typical shouldered survival curve was apparent (**Figure 1A**). There was no difference in survivability of *H. marinus* if the shock experiments were performed at ambient room temperature, or at the optimal growth temperature of 65°C. Interestingly, concentrations of Na-perchlorate (500 mM), that is above the growth limit of *H. marinus* (440 mM; see paragraph below), did not lead to a reduction in survivability during long term exposure experiments. Only exposure to 1 M Na-perchlorate for 96 h reduced the survival rate by approximately two orders of magnitude.

For comparison, the survivability of three other, well investigated microorganisms originating from very different environments, namely *E. coli*, *D. radiodurans*, and *B. subtilis* (as vegetative cells or pure spores) was investigated after short term shock exposure to Na-perchlorate (**Table 1**). Differences in sensitivity were obvious (**Figure 1B**): *E. coli* was the most sensitive as determined by the survival curve as well by the calculated *D*_10_-value (**Table 1**). Exposure to 2.5 M Na-perchlorate for 15 min reduced the survival of *E. coli* by more than four orders of magnitude. Higher concentrations led to complete loss of viability. Vegetative *B. subtilis* cells showed a medium sensitivity against short term shock exposure to Na-perchlorate and were completely inactivated by concentrations higher than 4 M Na-perchlorate. *D. radiodurans* also reacted significantly to an exposure (15 min) to Na-perchlorates, and exposure of 4 M Na-perchlorate led to a reduction in survivability by three orders of magnitude. Only purified spores were as resistant as *H. marinus* cells and did not react after a treatment with Na-perchlorate up to 5 M (**Table 1**).

**Table 1:** Calculated *D*_10_-values of *H. marinus* and reference organisms after treatment with Na-perchlorate.

Strains	*D*_10_-value (15 min; NaClO_4_)
*H. marinus*	>5 M^a^
*B. subtilis* (purified spores)	>5 M^a^
*B. subtilis* (vegetative cells)	1.9 M
*E. coli*	1.3 M
*D. radiodurans*	2.7 M


*^a^Not determinable precisely.*

**FIGURE 2:**
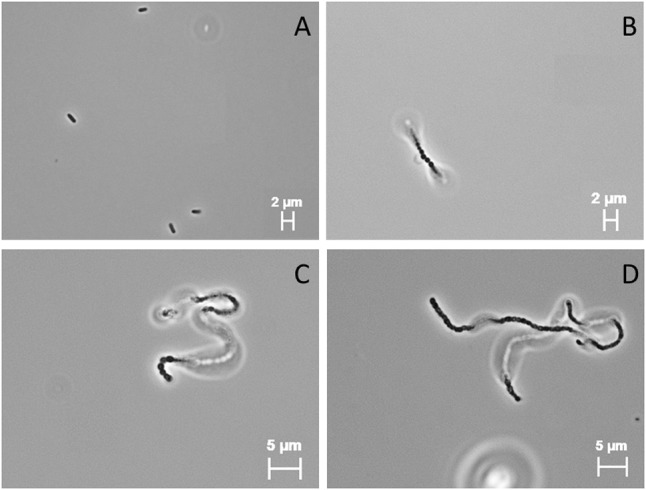
Light microscopic (100×; PH3) images of *H. marinus* under standard cultivation conditions **(A)** and cultivation in the presence of 300 mM Na-perchlorate **(B–D)**.

*Hydrogenothermus marinus* was able to replicate in the presence of 440 mM Na-perchlorate. Higher concentration (≥460 mM) led to an inability to replicate. However, the addition of Na-perchlorate during growth led to morphological changes. Under optimal growth conditions *H. marinus* cells usually appear as single motile short rods (**Figure 2A**); in the presence of Na-perchlorate chain formation in combination with a rounding of the single cells within the chain occurred in a concentration dependent manner. The threshold value below which no chain formation could be observed was 100 mM. At concentrations between 100 and 200 mM beside a minor fraction of single cells, short chains up to approximately 10 cells dominated. Concentrations between 200 and 440 mM led exclusively to long almost immobile chains with different lengths up to approximately 80 cells (**Figures 2B–D**). These morphological changes were reversible after the cells were transferred into media and cultivated overnight without perchlorate.

**FIGURE 3:**
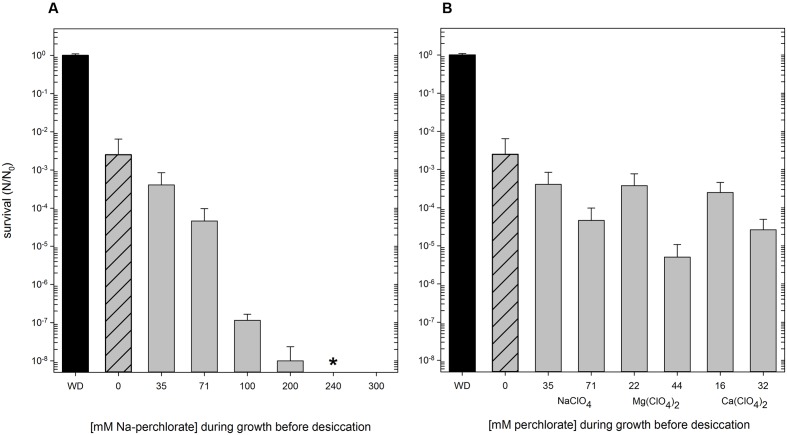
Survival of *H. marinus* after desiccation (24 h, oxic conditions), preceding cultivation of the cells was carried out at the indicated concentrations of perchlorates. Reactivation after desiccation was carried out in standard (perchlorate free) medium. Ascending concentration of Na-perchlorate up to 300 mM **(A)**. Different concentration of different perchlorates [NaClO_4_, Mg(ClO_4_)_2_, Ca(ClO_4_)_2_] corresponding to 0.5% (wt/vol) and 1.0% (wt/vol), respectively **(B)**. WD: without desiccation; shaded: no perchlorate during cultivation; ^∗^ no viable cells could be detected after reactivation.

*Hydrogenothermus marinus* is tolerant to desiccation under oxic and anoxic conditions is the same way (Beblo et al., 2009). Nevertheless, the desiccation tolerance was negatively influenced by the presence of different perchlorates. Below 100 mM Na-perchlorate during cultivation, only minor effects occurred. In contrast, concentrations of 100 mM and higher had an important influence on desiccation tolerance. Even if *H. marinus* is able to grow in the presence of Na-perchlorate up to 450 mM, perchlorate treated cells showed survival after desiccation only up to a maximum of 200 mM Na-perchlorate [S (24 h, 200 mM NaClO_4_) = 1 × 10^-8^] (**Figure 3A**).

The presence of Mars analogous concentrations (0.5% wt/vol and 1.0% wt/vol, corresponding to 35 mM and 71 mM NaClO_4_) of different perchlorates (Na-, Mg-, and Ca-perchlorate) during growth also influenced the survival after desiccation treatment. The type of cation of the perchlorate did not have a significant effect on the survival after desiccation. After 24 h of desiccation under oxic conditions, perchlorates in concentration of 0.5% (wt/vol) led to an average survival rate of 3 × 10^-4^; concentrations of 1% (wt/vol) entail an additional reduction of the survival rate of one to two orders of magnitude (**Figure 3B**).

## Discussion

Halophilic Archaea and Bacteria (e.g., *Halobacterium salinarum*; *Halomonas elongata*) are able to grow to some extent in perchlorate containing medium up to 0.4 M Na-perchlorate (Oren et al., 2014; Matsubara et al., 2017). Compared to this, *H. marinus* was as tolerant as the halophilic strains to perchlorates and could grow in the presence of 0.45 M Na-perchlorate. Similar results were obtained for the hyperthermophilic Archaeon *A. fulgidus* where growth was observable up to 0.4 M Na-perchlorate. During the metabolic studies with *A. fulgidus* it could be shown that perchlorates are stable at different cultivation conditions: for example, incubation temperatures of 85°C and high salt concentrations do not lead to a degradation of the perchlorates during cultivation (Liebensteiner et al., 2013; Al Soudi et al., 2017). Therefore, degradation effects for of *H. marinus*, with an incubation temperature of 65°C at moderate salt conditions can be excluded.

The extraordinarily high tolerance of *H. marinus* was obvious in the short term shock exposure experiments where the exposure concentration exceeded by far the concentration where growth is possible: 15 min in 5 M Na-perchlorates at room temperature did not lead to any change in survivability. Only 96 h of exposure resulted in a reduced survivability. The Archaea *Methanothermobacter wolfeii*, and *Methanosarcina barkeri* were able to survive exposure to 5 and 25% perchlorates (Mg-, Ca-, Na-perchlorate) for variable lengths of time with *M. wolfeii* surviving the 25% (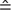 1.1 M Mg-perchlorate) concentration for 72 h (Kral et al., 2016).

In general, modifying size and shape in response to changes in the environmental conditions or to stress factors is known. Filamentation, based on an incomplete cell division process, is one observed shape alteration that can be influenced by several factors such as nutrient deprivation, oxidative stress, DNA damage, exposure to antibiotics and temperatures shifts (Young, 2006; Justice et al., 2008; Perfumo et al., 2014). The presence of perchlorates during growth led to a clearly visible change in *H. marinus*’ morphology. At concentrations exceeding 0.2 M, chain formation was observable. When Na-perchlorate was no longer present, the morphological changes were completely reversible and the cells were growing similar to control cells. It seemed that Na-perchlorate triggers the chain formation. Morphological changes due to Na-perchlorate are also described for halophilic and methanogenic microorganisms. Grown at the highest tolerated perchlorate concentrations, *H. salinarum*, *Haloferax mediterranei*, and *Haloarcula marismortui* were unusually swollen and deformed. For instance, *H. elongata* cells looked normal up to 0.2 M Na-perchlorate, but in a medium with 0.4 M Na-perchlorate, the cells had a thin and wrinkled appearance (Oren et al., 2014). One methanogenic strain, *Methanobacterium arcticum*, shows other morphological changes and builds cyst-like cells in the presence of Mg-perchlorate (Shcherbakova et al., 2015). The reason for the morphological change of *H. marinus* is not known. It could be speculated that the chain formation provides an advantage in the survival during cell damaging conditions. Comparable phenomena, including cell aggregation and biofilm formation have already been observed in *Sulfolobus solfataricus* after UV-irradiation and in *A. fulgidus* in various stress conditions (LaPaglia and Hartzell, 1997; Fröls et al., 2008). In addition to these two examples for hyperthermophilic Archaea, filament formation is also known in Bacteria such as *Listeria monocytogenes* and *Campylobacter jejuni* (Cameron et al., 2012; Jones et al., 2013). In these instances the filamentation is caused by sublethal (e.g., hyperosmotic) stress.

The cellular mechanisms for the morphological changes in *H. marinus* remain speculative. It seemed that the perchlorates ions penetrated the cells and interfere with the cell metabolism. The cells were not able to divide properly in the presence of Na-perchlorate. Which specific part of the cell division mechanism is influenced by perchlorates cannot be clarified so far: possible targets could be proteins used by microorganisms for septum formation and cell separation/division. In this process at least nine proteins (eight Fts proteins and the ZipA protein) are involved (Lutkenhaus and Addinall, 1997). For example, mutants of *E. coli* which have a mutation in the FtsA or the FtsZ protein grow in filaments instead of single cells (Walker et al., 1975; Addinall et al., 1996). Comparable results were obtained for *E. coli* ZipA mutants (Pichoff and Lutkenhaus, 2002). Since the genome sequence and a knock out mutant system for *H. marinus* are lacking, we could not elucidate which proteins are affected, a situation also found for the other microorganisms with morphological changes like *H. salinarum*, *H. mediterranei, H. marismortui*, and *H. elongata*.

Not only morphology was influenced by the presence of perchlorates, but also *H. marinus’* desiccation tolerance was affected by perchlorates. Compared to other vegetative cells, *H. marinus* shows a significant desiccation tolerance and can survive periods of water loss for up to 6 months (Beblo et al., 2009). The combination of exposure to perchlorates during growth followed by desiccation led to a typical additive effect. The additive model may be particularly appropriate when stressors affect different physiological processes (Folt et al., 1999). However, it seemed that only single cells and not the cell chains were tolerant to desiccation. Lower perchlorate concentrations (0.5%; 1%) leading to a mixture of chains and single cells resulted in a reduced survivability after desiccation. Concentrations higher than 0.2 M, lead exclusively to growth in chains, resulted in a complete disappearance of desiccation tolerance.

In general, the question arises whether the perchlorate ions themselves and/or the corresponding counter ions play a role in affecting the cells. Previous studies showed that *H. marinus* is very tolerant to high salinity. The addition of NaCl (up to 1.2 M) during growth leads to formation and accumulation of compatible solutes and an elevation of desiccation tolerance in *H. marinus* (Beblo-Vranesevic et al., 2017). For some halotolerant strains, an influence of the counter ions can be neglected (Al Soudi et al., 2017). For *H. marinus* an influence of the counter ions (Na^+^, Ca^2+^, Mg^2+^) can nearly be excluded, because all of these ions are also present in the standard cultivation medium (VM-1). VM-1 medium contains 0.4 M of Na^+^ ions, 0.13 M of Mg^2+^ ions and 0.01 M of Ca^2+^ ions (Stöhr et al., 2001). These ions are present in lower concentration in the standard medium than during exposures to the corresponding perchlorates in the experiment described above. Nevertheless, *H. marinus* is not only adapted to them and is even dependent on their presence.

On Mars, liquid water may be temporarily available within brines. Perchlorates, as hydroscopic substances, bind water from the atmosphere and contribute to the formation of these brines, which remain liquid at low temperatures prevailing on the surface of Mars (Gough et al., 2011; Toner and Catling, 2016). Martian brines contain high amounts of different dissolved salts, including chlorides, sulfates, and perchlorates (Kounaves et al., 2014; Fox-Powell et al., 2016). Within this transient liquid water on the surface of Mars, a simultaneous appearance of high salt concentrations and perchlorates occur (Martín-Torres et al., 2015). In subsurface of Mars pressure and temperature increases with depth and thereby the probability of liquid, additionally radiation protected, water reservoirs (such as subsurface aquifers) rises, too (Boston et al., 1992; Dartnell et al., 2007). Thinking about Martian habitability these areas could be one option where microbial life could be preserved, survive or even thrive.

In general, *H. marinus* unites some properties which are of essential importance when discussing the past and present habitability of Mars: the organisms can only grow at low oxygen concentrations (down to 0.5 vol % O_2_, Stöhr et al., 2001), the Martian atmosphere contains today an average oxygen concentration of 0.13% (Horneck, 2000); the cells are able to grow in the presence of Martian concentrations of perchlorates in combination with an exceptional desiccation tolerance (Beblo et al., 2009); they are tolerant to salt concentrations up to 1.2 M (Stöhr et al., 2001; Beblo-Vranesevic et al., 2017); the organism shows survival after exposure to UV-C and ionizing radiation (Beblo et al., 2011). The radiation dose rate of ionizing radiation on the surface of Mars was measured and calculated to be up to 0.21 mGy per day (Hassler et al., 2014; Matthiä et al., 2016) and is therefore significantly lower than in the applied experiments. In these experiments, viable cells could still be found after a dose of 5 kGy (Beblo et al., 2011). It is assumed that even radiation sensitive organisms, such as *H. marinus*, could survive the radiation occurring on the Martian surface or in the first centimeter of Martian regolith for several years or decades (Dartnell et al., 2007). Altogether, this makes *H. marinus* a promising new model organism for studies in Astrobiology.

## Author Contributions

KB-V and Kathya Bustamante (KB) performed experiments. KB-V wrote the manuscript. HH and PR contributed to the design of the study and both contributed to the writing and editing of the manuscript.

## Conflict of Interest Statement

The authors declare that the research was conducted in the absence of any commercial or financial relationships that could be construed as a potential conflict of interest.
